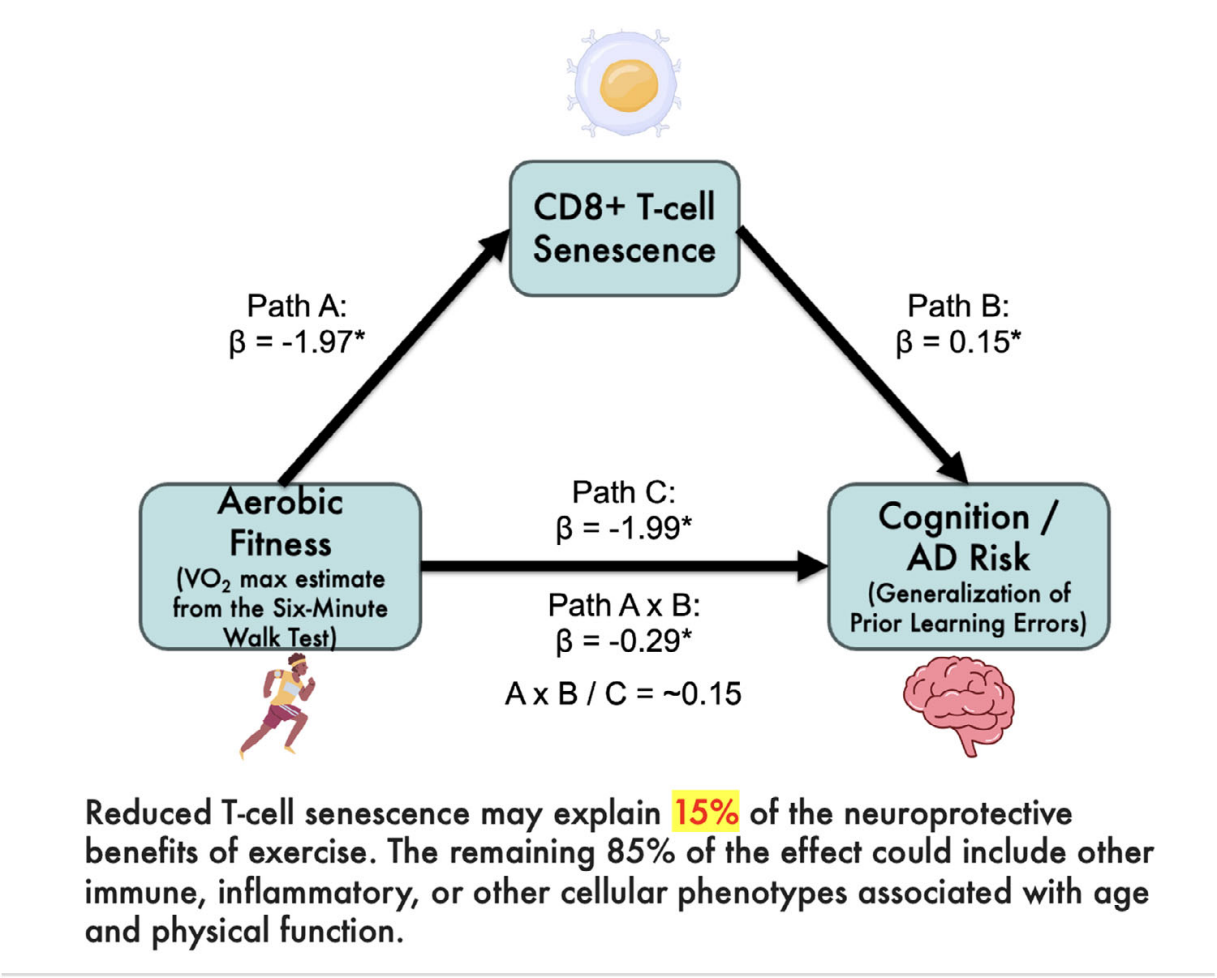# Lower CD8+ T‐cell Senescence Partially Mediates the Neuroprotection of Higher Aerobic Fitness

**DOI:** 10.1002/alz70856_107391

**Published:** 2026-01-11

**Authors:** Bernadette A. Fausto, Elizabeth Akbulut, Mustafa Sheikh, Stephanie Ghaly, Alicia Codrington, Andrew Gamil, Imran Arshad, Darian A. Napoleon, Robert Perna, Wiktoria Piaszczynska, Mark A. Gluck, Patricia Fitzgerald‐Bocarsly

**Affiliations:** ^1^ Rutgers University–Newark, Newark, NJ, USA; ^2^ Rutgers New Jersey Medical School, Newark, NJ, USA

## Abstract

**Background:**

Immunosenescence––age‐related changes in immunity––may exacerbate the pathologic processes of Alzheimer's disease. Compartments of the *adaptive* immune system (e.g., cytotoxic CD8+ T‐cells) show the most significant decline in later life. Fortunately, a higher level of aerobic fitness is linked to both lower age‐related accumulation of senescent T‐cells and reduced Alzheimer's risk. However, it remains unclear whether this association between higher aerobic fitness and decreased Alzheimer's risk is mediated by lower proportions of T‐cell senescence. In a cohort of older African Americans, this study aimed to: 1) examine the relationship between aerobic fitness and generalization (a sensitive cognitive marker of Alzheimer's risk) and 2) investigate whether CD8+ T‐cell senescence mediates this relationship.

**Method:**

231 older African American participants from the *Pathways to Healthy Aging in African Americans* cohort study (*M*_age_=70.74 years, *SD* = 6.40; *M*_education_=14.02 years, *SD* = 2.25) responded to demographic, health, and lifestyle questionnaires; completed a cognitive battery including a generalization task (Concurrent Discrimination and Transfer Task); underwent an anthropometric and physical performance battery; and provided a blood sample for T‐cell senescence characterization. Using the blood specimens, peripheral blood mononuclear cells were isolated and analyzed for senescence‐associated ß‐Galactosidase activity as a measure of proportions of cytotoxic CD8+ T‐cell senescence. Aerobic fitness (VO_2_peak) was estimated from the Six‐Minute Walk Test. Covariates included age, sex, education, and waist‐to‐hip ratio.

**Result:**

Higher aerobic fitness was significantly associated with fewer generalization errors. The direction of the paths indicated that higher aerobic fitness was associated with lower CD8+ T‐cell senescence ( = ‐0.29, *p* = .03), which was subsequently associated with a decrease in generalization errors ( = 0.15, *p* = .01). Overall, reduced T‐cell senescence may explain 15% of the neuroprotective benefits of exercise.

**Conclusion:**

One pathway by which higher aerobic fitness is associated with lower Alzheimer's risk in older African Americans is through lower proportions of CD8+ T‐cell senescence. The remaining 85% of the effect could include other immune, inflammatory, or other cellular phenotypes associated with age and physical function. These results highlight the immune and cognitive function benefits of a physically active lifestyle.